# T1 mapping and T2 mapping at 3T for quantifying the area-at-risk in reperfused STEMI patients

**DOI:** 10.1186/s12968-015-0173-6

**Published:** 2015-08-12

**Authors:** Heerajnarain Bulluck, Steven K. White, Stefania Rosmini, Anish Bhuva, Thomas A. Treibel, Marianna Fontana, Amna Abdel-Gadir, Anna Herrey, Charlotte Manisty, Simon M. Y. Wan, Ashley Groves, Leon Menezes, James C. Moon, Derek J. Hausenloy

**Affiliations:** The Hatter Cardiovascular Institute, Institute of Cardiovascular Science, University College London, London, WC1E 6HX UK; The National Institute of Health Research University College London Hospitals Biomedical Research Centre, London, UK; The Heart Hospital, University College London Hospital, London, UK; UCL Institute of Nuclear Medicine, University College London Hospital, London, UK; Cardiovascular and Metabolic Disorders Program, Duke-National University of Singapore, Singapore, Singapore; National Heart Research Institute Singapore, National Heart Centre Singapore, Singapore, Singapore

**Keywords:** Area-at-risk, Myocardial salvage, Cardiovascular MR, T1 mapping, T2 mapping, ST-elevation myocardial infarction, Primary percutaneous intervention

## Abstract

**Background:**

Whether T1-mapping cardiovascular magnetic resonance (CMR) can accurately quantify the area-at-risk (AAR) as delineated by T2 mapping and assess myocardial salvage at 3T in reperfused ST-segment elevation myocardial infarction (STEMI) patients is not known and was investigated in this study.

**Methods:**

18 STEMI patients underwent CMR at 3T (Siemens Bio-graph mMR) at a median of 5 (4–6) days post primary percutaneous coronary intervention using native T1 (MOLLI) and T2 mapping (WIP #699; Siemens Healthcare, UK). Matching short-axis T1 and T2 maps covering the entire left ventricle (LV) were assessed by two independent observers using manual, Otsu and 2 standard deviation thresholds. Inter- and intra-observer variability, correlation and agreement between the T1 and T2 mapping techniques on a per-slice and per patient basis were assessed.

**Results:**

A total of 125 matching T1 and T2 mapping short-axis slices were available for analysis from 18 patients. The acquisition times were identical for the T1 maps and T2 maps. 18 slices were excluded due to suboptimal image quality. Both mapping sequences were equally prone to susceptibility artifacts in the lateral wall and were equally likely to be affected by microvascular obstruction requiring manual correction. The Otsu thresholding technique performed best in terms of inter- and intra-observer variability for both T1 and T2 mapping CMR. The mean myocardial infarct size was 18.8 ± 9.4 % of the LV. There was no difference in either the mean AAR (32.3 ± 11.5 % of the LV versus 31.6 ± 11.2 % of the LV, *P* = 0.25) or myocardial salvage index (0.40 ± 0.26 versus 0.39 ± 0.27, *P* = 0.20) between the T1 and T2 mapping techniques. On a per-slice analysis, there was an excellent correlation between T1 mapping and T2 mapping in the quantification of the AAR with an R^2^ of 0.95 (*P* < 0.001), with no bias (mean ± 2SD: bias 0.0 ± 9.6 %). On a per-patient analysis, the correlation and agreement remained excellent with no bias (R^2^ 0.95, *P* < 0.0001, bias 0.7 ± 5.1 %).

**Conclusions:**

T1 mapping CMR at 3T performed as well as T2 mapping in quantifying the AAR and assessing myocardial salvage in reperfused STEMI patients, thereby providing an alternative CMR measure of the the AAR.

## Background

Despite timely myocardial reperfusion by primary percutaneous coronary intervention (PPCI), patients presenting with an acute ST-segment elevation myocardial infarction (STEMI) still experience significant morbidity and mortality [1–3]. New cardioprotective therapies are therefore required to reduce myocardial infarct (MI) size, in order to preserve left ventricular (LV) ejection fraction and prevent the onset of heart failure. The assessment of the efficacy of novel cardioprotective therapies requires the accurate quantification of the area-at-risk (AAR), as this enables the measurement of the myocardial salvage index (AAR subtract MI size/AAR), a more sensitive measure of cardioprotective effectiveness than a reduction in absolute MI size or MI size as a percentage of the LV alone [4, 5].

In this regard, T2-weighted (short tau inversion recovery) cardiovascular magnetic resonance (CMR) of myocardial edema in the first few days following PPCI has emerged as a promising technique for retrospectively quantifying the AAR in reperfused STEMI patients [6, 7]. This approach, however, does have its limitations which include a low contrast-to-noise ratio, susceptibility to blood pool and motion artifacts, and signal drop-out. Some of these can be overcome using T2-mapping CMR, whch has emerged as a more robust surrogate marker to delineate the AAR in this setting [8, 9]. However, recent studies have found that in addition to reducing MI size, certain cardioprotective therapies such as ischemic postconditioning [10] and remote ischemic conditioning [11, 12] also decreased the extent of myocardial edema as delineated by T2 mapping and T2-weighted CMR, resulting in an underestimation of the AAR with this approach.

Recently, *native* T1-mapping CMR (referred to as T1-mapping or T1 map throughout the manuscript for simplicity) has been found to be superior to T2-weighted CMR in detecting myocardial edema in the context of acute myocarditis [13] and acute myocardial infarction [14]. T1-mapping CMR has recently been reported to accurately quantify the AAR in the canine heart subjected to acute myocardial infarction [15]. Langhans et al. [16] found that AAR by T1 and T2 mapping CMR at 1.5 T correlated well with that obtained with myocardial SPECT. In healthy volunteers, von Knobelsdorff-Brenkenhoff et al. [17] have recently demonstrated feasibility and provided reference values for T1 and T2 mapping at 3T, but the role of these CMR sequences at 3T has not been investigated in the setting of acute MI. Whether T1-mapping CMR can quantify the AAR at 3T, to our knowledge, has not been directly compared to T2 mapping in reperfused STEMI patients, and was investigated in the current study.

## Methods

### Patient population

18 PPCI-treated STEMI patients were recruited over a 7-month period from one UK center. The main exclusion criteria were previous MI and standard contraindications to CMR (significant claustrophobia, severe allergy to gadolinium chelate, estimated glomerular filtration rate <30 mL/min/1.73 m^2^, presence of ferromagnetic implants). All eligible patients provided informed written consent and the local ethics committee (London - Harrow) approved all study-related procedures. The patients recruited in this study formed part of a cohort of patients included in a recently conducted hybrid simultaneous Positron Emission Tomography/MR study [18].

### CMR

Patients underwent CMR at a median of 5 (4–6) days post-PPCI using a 3T MR scanner (Biograph mMR; Siemens Healthcare, Erlangen, Germany). The MR imaging protocol included cine imaging for function, followed by T1 maps and T2 maps for AAR and Late Gadolinium Enhancement (LGE) for MI size. T1 maps and T2 maps (Works in Progress, software #699, Siemens Healthcare, Frimley, UK) were acquired as previously described [17]. For T1 maps by SSFP-based Modified Look-Locker Inversion Recovery (MOLLI) technique, imaging parameters were: repetition time = 2.6−2.7 ms; echo time = 1.0−1.1 ms; flip angle = 35°; matrix = 256x144; slice thickness 6 mm. Images were acquired at different inversion times (5-3-3 modified MOLLI protocol to reduce heart rate variability by acquiring 5 images after the first inversion pulse, followed by a 3 heartbeat pause and then acquiring the last 3 images after the second inversion pulse [19]) and registered prior to a non-linear least-square curve fitting to generate a pixel-wise coloured T1 map. For T2 maps, 3 single shot images were acquired at different T2-preparation times (0 ms, 24 ms, and 55 ms, respectively) and imaging parameters were: repetition time = 2.4 ms; echo time = 1 ms; flip angle = 70°; repetition time = 3× R-R interval; acquisition matrix 116×192; slice thickness 6 mm; field of view adjusted as per subject size. Motion correction and fitting were performed to estimate coefficients of the decay function, which were then used to estimate the T2 times. An in-built specific colour look-up table was then used to derive the coloured T2 maps.

**Fig. 1:**
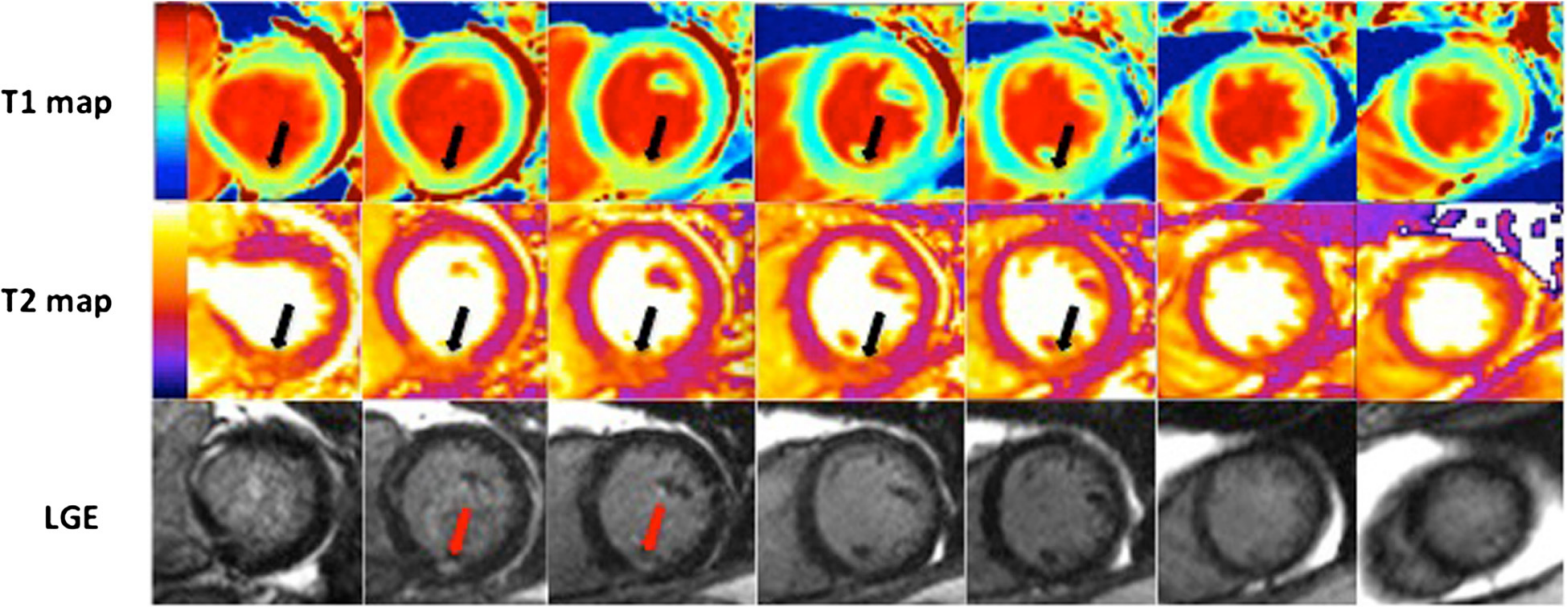
Matching T1 maps, T2 maps and LGE short axis CMR images from base to apex of a patient presenting with an acute inferior STEMI reperfused by PPCI. Both T1 and T2 maps delineate the AAR (black arrows) and the LGE images show a small subendocardial myocardial infarct (red arrows)

The T1 and T2 maps were acquired to match the short-axis cines to cover the entire left ventricle. Gadolinium contrast (Gadoterate meglumine, gadolinium-DOTA, marketed as Dotarem, Guerbet S.A., Paris, France) was administered at a dose of 0.1 mmol/kg. LGE was performed using segmented two-dimensional inversion-recovery turbo fast low-angle shot LGE sequences (repetition time = 864 ms; echo time = 1.56 mm; acquisition matrix = 123×256; inversion time = 400 – 500 ms, flip angle = 20°; slice thickness 8 mm) 10–15 min after single bolus contrast agent injection and short-axis slices of the entire left ventricle were acquired to match the T1 maps and T2 maps (Fig. 1).

### Imaging analysis

Quantification of LV volumes, LV mass, LV ejection fraction and MI size were performed using CVI42 software (Version 5.1.0[280], Calgary, Canada). MI size was quantified following manual delineation of the endocardium and epicardium of short axis slices and using 5 standard deviations (SD) threshold above the mean remote myocardium [20]. Transmural extent of LGE was also quantified using 100 chords for each short axis slice and averaging the mean transmural extent for each segment using the modified 16 segment American Heart Association (AHA) model [21]. Segments were assigned an LGE score of either “0” for no LGE and “1” for the presence of LGE. Mean segmental T1 and T2 values were also automatically generated for the modified 16 segment AHA model using CVI42 after manually drawing the endocardial and epicardial borders on all short axis slices. The time taken to acquire the 2 mapping sequences for each patient (LV coverage from base to apex) were recorded. AAR by T1 mapping and T2 mapping were quantified using an in-house macro written in ImageJ (Version 1.45 s, National Institute of Health, USA). The endocardium and epicardium borders of matching LV short axis T1 maps and T2 maps were manually segmented (excluding the papillary muscles) to obtain the myocardium volume. Two experienced observers performed all subsequent quantification on the pre-segmented images blindly and independently, and one of the observers performed the analysis twice, 3 months apart. The affected myocardium on the T1 maps and T2 maps were quantified using 3 analytical techniques namely manual delineation, 2SD above the mean remote normal myocardium, and the automated Otsu detection method (Otsu technique) [22]. In brief, the Otsu technique uses an algorithm to automatically divide the signal intensity histogram into normal and enhanced. An exhaustive search for values that minimize the intraclass variance between two populations of signal intensities is used to establish the threshold [23]. Analysis was performed for all the slices for each patient to obtain the “enhanced” myocardium as a percentage of the whole LV. Regions-of-interest were drawn within the AAR (avoiding areas of MVO), and within the remote myocardium to obtain representative T1 and T2 values at 3T CMR. Manual correction was performed for areas of pseudonormalization within the MI zone (corresponding to areas of MVO) and areas of hyperenhancement due to any obvious blood pool or pericardial partial voluming and off-resonance artifacts in the remote myocardium. All slices were visually assessed by the 2 experienced observers and those with significant partial voluming and susceptibility or motion artifacts overlapping with the affect myocardium were excluded by consensus. In cases of doubt or disagreement, the raw images were used to decide whether that slice was to be excluded or not as previously described by von Knobelsdorff-Brenkenhoff et al. [17].

### Statistical analysis

Statistical analysis was performed using SPSS (Version 22, IBM Corporation, Illinois, USA) and MedCalc (Version 15.6.1, MedCalc Software bvba, Ostend, Belgium). Continuous data was expressed as mean ± SD or median (interquartile range). Categorical data was reported as frequencies and percentages. Both per-slice and per-patient comparison was performed. Paired student t-tests and Wilcoxon Rank sum test were performed to compare mean or median between paired groups. Pearson’s correlation coefficient expressed as its square (R^2^) was used to assess inter-method correlation. A linear regression analysis was performed to obtain the regression slope and its 95 % confidence interval to compare against the reference line with a slope of 1 which would represent AAR by T1 and T2 to be identical. Bland-Altman analysis was performed to assess agreement and bias detection between methods and presented as average difference ± 2SD. Inter-observer and intra-observer variability was assessed using intra-class correlation coefficient (ICC) and mean difference between interobserver and intraobserver measurements of T1 and T2 AAR ± SD. Receiver Operating Characteristic (ROC) analysis was performed to provide cut-off values for T1 and T2 for detecting acute myocardial necrosis defined by an LGE score of 1. ROC curves were compared for statistical difference using the method described by Delong et al. [24]. All statistical tests were two-tailed, and P-values of less than 0.05 were considered statistically significant.

## Results

**Fig. 2:**
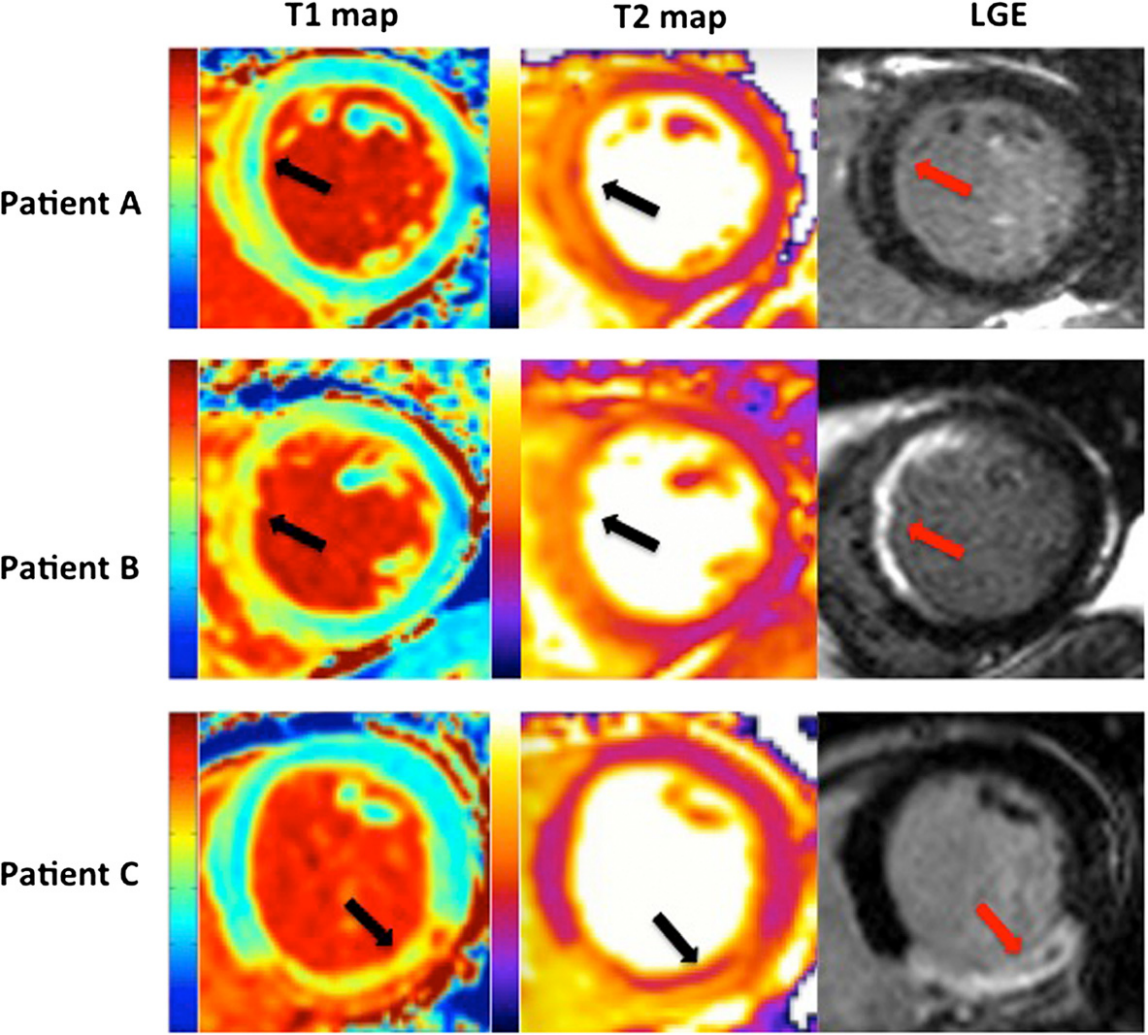
Representative mid left ventricular short axis T1 maps, T2 maps and LGE short-axis images from three patients demonstrating varying degrees of myocardial salvage. In patient A, both the T1 and T2 maps delineate a large area of myocardial edema in the left anterior descending (LAD) territory (black arrow), corresponding to the AAR, with no significant myocardial infarct on LGE image (red arrow), indicating complete myocardial salvage. In patient B, the T1 and T2 maps again delineate an area of myocardial edema in the LAD territory (black arrow), with a subendocardal myocardial infarct on the LGE image (red arrow), indicating some myocardial salvage. In patient C, the T1 and T2 maps delineate an area of myocardial edema in the right coronary artery territory (black), with a transmural myocardial infarct containing some microvascular obstruction (hypoenhancement on T2 map and LGE images) on the LGE image (red arrow), indicating minimal myocardial salvage

The patients’ demographics, coronary angiographic and CMR characteristics are detailed in Table 1. 83 % were male and the mean age was 58 ± 10 years. Two thirds of the patients presented with a left anterior descending (LAD) territory STEMI and one third with a right coronary artery (RCA) territory infarct. The median onset to balloon time was 292 (116 – 800) minutes. Figure 2 shows examples of T1 maps, T2 maps and LGE in 3 different patients.

**Table 1:** Patients’ demographics, coronary angiographic and CMR characteristics. This table provides the demographic details, coronary angiographic and CMR characteristics of the study population

Details	Number
Number of patients	18
Male (%)	15 (83)
Age ± SD (years)	58 ± 10
Hypertension (%)	5 (28)
Smoking (%)	9 (50)
Dyslipidemia (%)	3 (17)
Chest pain onset to balloon time (minutes)	292 (116–800)
Infarct artery and location (%)	
LAD	12 (67)
Proximal/ Mid/ Distal	5 (42)/6 (50)/1 (8)
RCA	6 (33)
Proximal/ Mid/ Distal	3 (50)/2 (33)/1(17)
Pre-PPCI TIMI flow (%)	
0/1/2/3	13 (72)/4 (22)/1 (6)/0 (0)
Post-PPCI TIMI flow (%)	
0/1/2/3	1 (6)/0 (0)/2 (12)/15 (82)
Single vessel disease	13 (72)
Double vessel disease	5 (28)
Days from PPCI to CMR	5 (4–6)
Left ventricular ejection fraction (%)	49 ± 11 (Normal range 58–76)
End diastolic volume (ml)	135 ± 21 (Normal range 113–196)
Left ventricular mass (g)	151 ± 50 (Normal range 107–184)
Presence of MVO (%)	8 (44)
Infarct size by LGE, (% LV volume)	18.8 ± 9.4
AAR by T1-mapping, (% LV volume)	32.3 ± 11.5
AAR by T2-mapping, (% LV volume)	31.6 ± 11.2

*PPCI* primary percutaneous coronary intervention, *LAD* left anterior descending artery, *RCA* right coronary artery, *TIMI* thrombolysis in myocardial infarction, *LV* left ventricle, *SD* standard deviation, *MVO* microvascular obstruction, *LGE* Late Gadolinium Enhancement, *AAR* area-at-risk

### Quality and acquisition times of T1 and T2 maps

In total, 125 pairs of T1 maps and T2 maps of the LV short axis from base to apex were acquired. The acquisition times for T1 maps and T2 maps per patient were similar (T1 maps: 5.6 ± 1.9 min, T2 maps: 5.5 ± 2.0 min, *P* = 0.89). 18 pairs of images were excluded due to suboptimal image quality (15 apical short-axis slices and 3 basal short-axis slices including the LVOT which had significant partial voluming). Both T1 and T2 CMR were equally prone to susceptibility artifacts in the lateral wall. As we did not have any patients with a circumflex territory infarction, these artifacts were remote from the AAR and the slices (10/107 slices, 9 %) were kept for analysis and manual correction was required. 27 slices had normal T1 and T2 values and 80 slices had abnormal T1 and T2 values (using 2SD from the remote myocardium as a reference). Both T1 and T2 maps were equally likely to be affected by MVO requiring manual correction to include the core as part of the AAR and occurred in 13/18 patients, 45/107 slices of the T2 maps, and 42/107 of the T1 maps.

### Inter and intra-observer variability

**Fig. 3:**
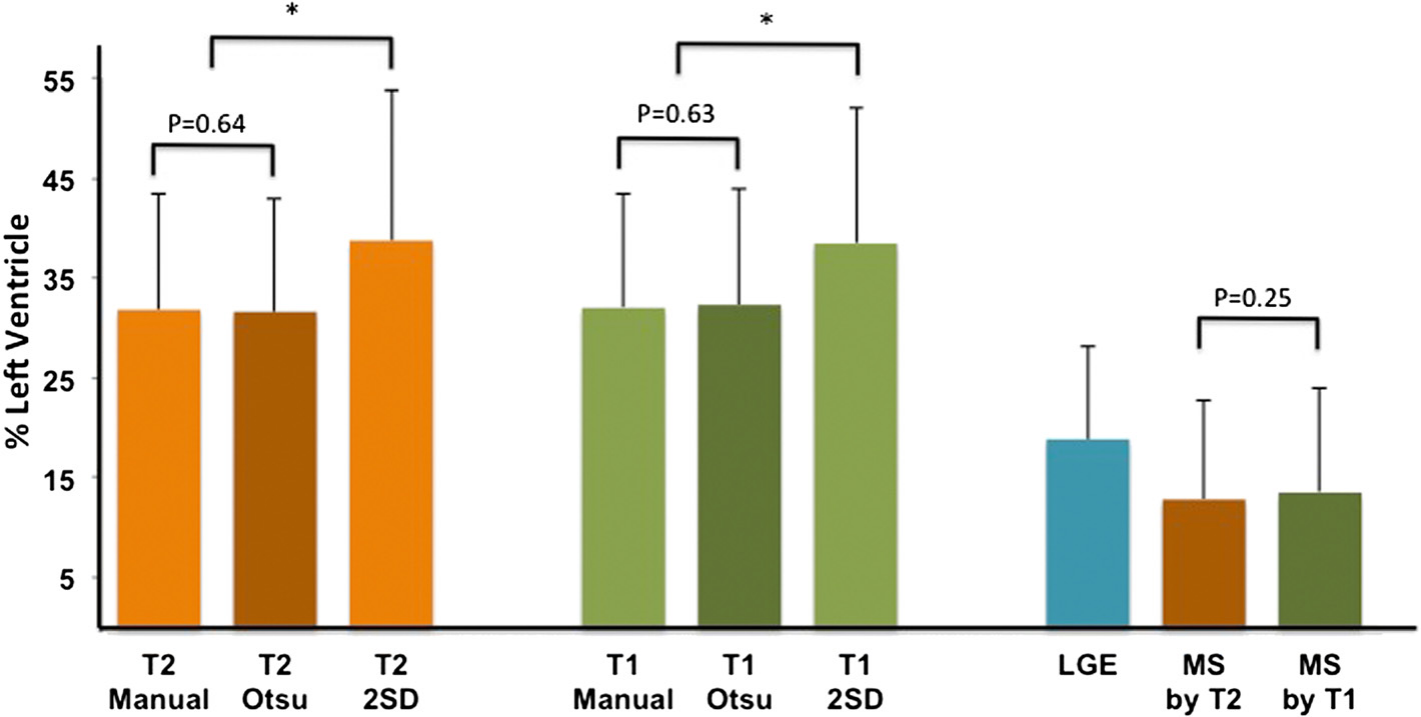
Performance of three different thresholding techniques for delineating the AAR on T1 and T2 maps. The AAR by the 2 standard deviation (2SD) technique was significantly larger than that delineated by the manual and Otsu thresholding techniques. There was no difference between the manual and Otsu techniques for both T1 and T2 mapping in delineating the AAR. *denotes significant statistical difference with *P* < 0.001

The AAR by the 3 techniques (manual/Otsu/2SD) for T2 mapping were 31.8 ± 11.7 %, 31.6 ± 11.2 % and 38.7 ± 15 % and for T1 mapping were 32.0 ± 11.5 %, 32.3 ± 11.5 % and 38.4 ± 13.6 % respectively. The ICC for intra-observer and inter-observer variability of the 3 analytical techniques were excellent, both for T1 and T2 mapping and was highest for the Otsu technique. The 2SD technique had the largest differences both for intra-observer and inter-observer measurements for both mapping techniques. These findings are summarized in Table 2. The 2SD technique overestimated the AAR compared to manual delineation (as the reference standard) but there was no difference between Otsu and manual delineation for both T1 and T2 mapping (Fig. 3). The Otsu derived T1 and T2 AAR was therefore used for the analysis below.

**Table 2:** Intra-observer and inter-observer variability of the area-at-risk by T1 and T2. Intra-observer and inter-observer variability of the area-at-risk by T1 and T2. This table provides the intra-class correlation coefficient (ICC) and mean difference ± SD for the inter-observer and intra-observer measurements of T1 mapping and T2 mapping using 3 analytical techniques for inter-observer and intra-observer variability

	ICC (95 % CI)	Mean difference ± SD (%)	P
Intra-observer variability (*n* = 107)			
T1 mapping			
Manual	0.961 (0.943 – 0.973)	1.5 ± 7.1	0.04*
2SD	0.948 (0.917 – 0.966)	2.6 ± 7.7	0.001*
Otsu	0.989 (0.984 – 0.993)	0.7 ± 3.2	0.03*
T2 mapping			
Manual	0.951 (0.928 – 0.966)	0.8 ± 6.4	0.18
2SD	0.965 (0.942 – 0.978)	2.4 ± 6.4	0.001*
Otsu	0.996 (0.995 – 0.998)	0.2 ± 1.9	0.24
Inter-observer variability (*n* = 107)			
T1 mapping			
Manual	0.980 (0.972 – 0.987)	0.3 ± 4.3	0.52
2SD	0.948 (0.925 – 0.964)	3.7 ± 7.2	0.001*
Otsu	0.993 (0.990 – 0.995)	0.2 ± 2.6	0.55
T2 mapping			
Manual	0.964 (0.947 – 0.975)	0.3 ± 6.1	0.59
2SD	0.960 (0.914 – 0.978)	3.5 ± 6.5	0.001*
Otsu	0.993 (0.989 – 0.995)	0.7 ± 2.6	0.008*

*denotes significant statistical difference with P  <  0.05

### Quantification of the AAR by T1 mapping

**Fig. 4:**
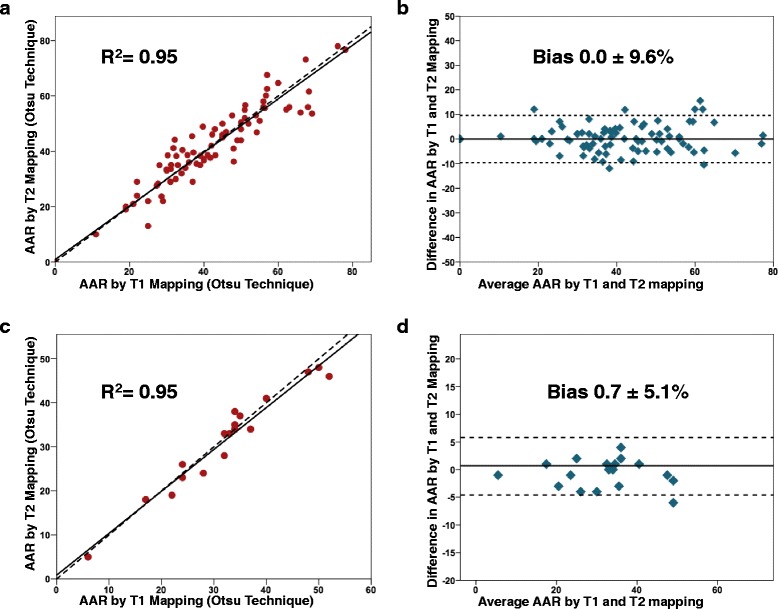
Correlation and agreement between T1 and T2 mapping to delineate the AAR. Both on a per-slice (**a** and **b**) and per-patient analysis (**c** and **d**), there was an excellent correlation and agreement between T1 and T2 mapping technique to delineate the AAR. The interrupted lines in a and c represent reference lines with a slope of 1

On a per-slice analysis, there was an excellent correlation between the T1 mapping and T2 mapping with an R^2^ of 0.95 and a regression slope 0.97 (95 % CI 0.93 – 1.01). There was no bias on Bland Altman analysis (mean ± 2SD: bias 0.0 ± 9.6 %) as illustrated in Fig. 4a. On a per-patient analysis, the correlation and agreement remained excellent with no bias (R^2^ 0.95, regression slope 0.95 (95 % CI 0.84 – 1.07), bias 0.7 ± 5.1 %; Fig. 4b). The mean AAR (expressed as a % of the LV) quantified by T1 mapping was similar to that by T2 mapping (32.3 ± 11.5 % of the LV, range 6 to 52 % by T1 mapping, versus 31.6 ± 11.2 % of the LV, range 5 - 48 % by T2 mapping, *P* = 0.25).

### MI size and myocardial salvage

The mean MI size by LGE was 18.8 ± 9.4 % of the LV, (range 2.0 - 34.0 %). Myocardial salvage (AAR subtract the MI size) was 12.8 ± 10.0 % of LV (range 0 – 42.0 %) by T2 mapping. The myocardial salvage index (myocardial salvage/AAR) was 0.40 ± 0.26 (range 0 – 0.89). There were no difference in either myocardial salvage (12.8 ± 10.0 % of LV by T2 mapping versus 13.5 ± 10.4 % of LV by T1 mapping, *P* = 0.25) (Fig. 3) or the myocardial salvage index (0.40 ± 0.26 by T2 mapping versus 0.39 ± 0.27 by T1 mapping, *P* = 0.20) between the 2 mapping techniques.

### T1 and T2 values in the AAR and remote myocardium

The T1 and T2 values in the AAR was significantly higher than those in the remote myocardium (T1 AAR: 1524 ± 116 ms, T1 remote myocardium: 1163 ± 78 ms, *P* < 0.001; T2 AAR: 72 ± 7 ms, T2 remote myocardium: 46 ± 3 ms, *P* < 0.001).

### Diagnostic performance of T1 and T2 mapping to detect acute myocardial necrosis

**Fig. 5:**
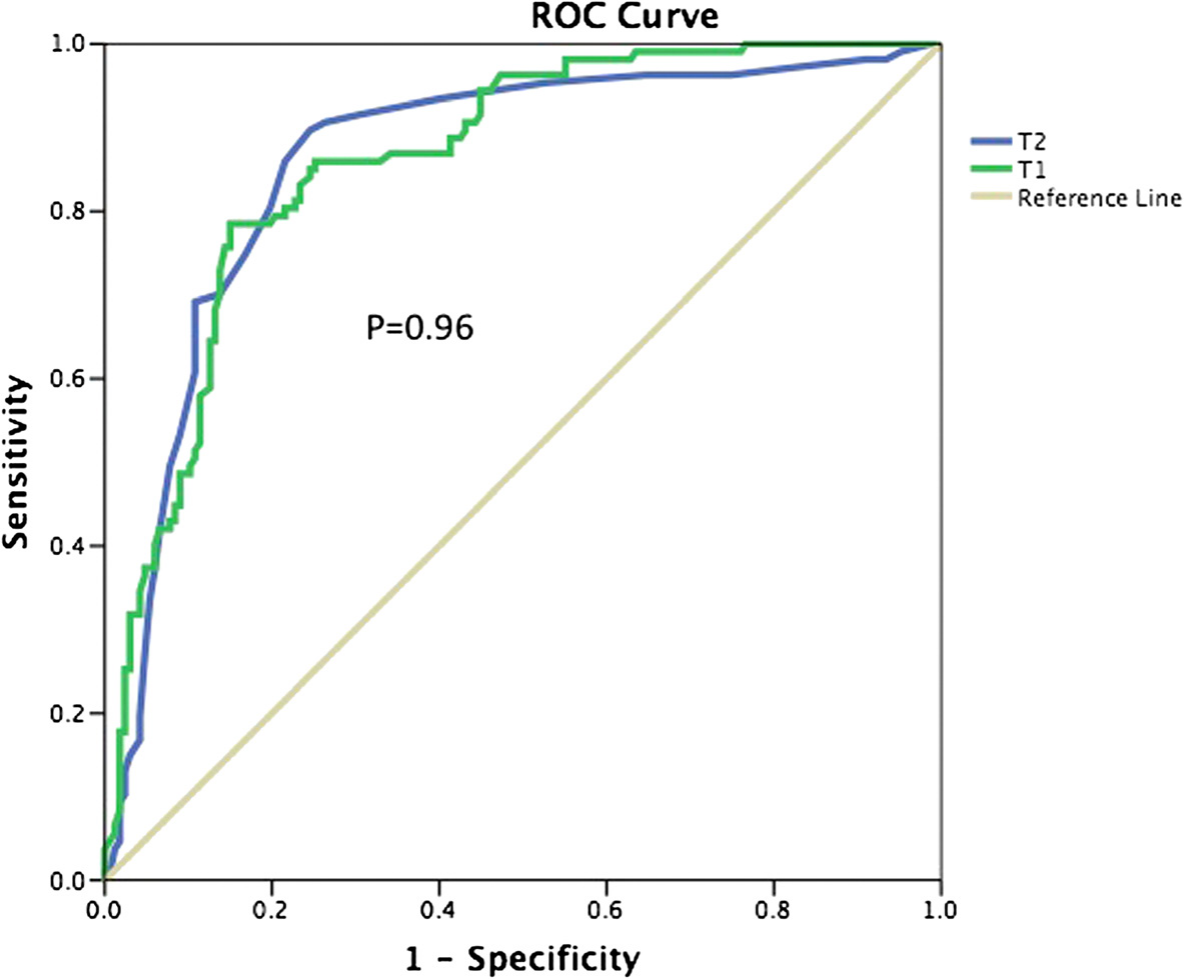
ROC curve showing the diagnostic performance of T1 mapping against T2 mapping to detect acute myocardial necrosis. Both T1 and T2 mapping performed equally well to detect acute myocardial necrosis. The AUC was 0.87 ± 0.02 for T1 and 0.86 ± 0.02 for T2, *P* = 0.96

Both T1 and T2 mapping performed well to detect acute myocardial necrosis delineated by the presence of LGE as illustrated in the ROC curves in Fig. 5. The area under the curve (AUC) was 0.87 ± 0.02 for T1 and 0.86 ± 0.02 for T2, *P* = 0.96. A T1 value of > 1249 ms had a sensitivity of 83 % and specificity of 80 % and a T2 value of > 52 ms had a sensitivity of 85 % and specificity of 82 % to detect acute myocardial necrosis defined by an LGE score of 1.

## Discussion

This is the first clinical study to demonstrate T1 mapping CMR at 3T can accurately quantify the AAR delineated by T2 mapping CMR in reperfused STEMI patients. There was an excellent correlation and agreement between T1 and T2 mapping in delineating the AAR. This data confirms the findings of the pre-clinical study by Ugander et al. [15] in the reperfused canine heart. Both T1 and T2 mapping CMR were equally prone to susceptibility artifacts at 3T and equally affected by MVO. There was no difference in the acquisition times on a per-patient basis.

Unlike the canine model from Ugander et al. [15], we included patients with different ischemic times (chest pain onset to PPCI times), different degrees of myocardial salvage, LAD as well as RCA infarcts, and with the presence of MVO. The current study therefore reinforces emerging evidence that T1 mapping performs as well as T2 mapping at 3T and therefore provides us with an additional tool to quantify the AAR.

This is the first study to compare the diagnostic performance of T1 mapping against T2 mapping at 3T to detect acute myocardial necrosis and the results are in keeping with the previous study by Dall’Armellina et al. [14] in which T1 mapping by ShMOLLI was compared with T2 weighted imaging at 3T. We performed an indirect comparison of the 2 ROC curves by the MOLLI protocol used in our study and ShMOLLI T1 mapping from their study [14] and found that there was no difference between the 2 AUCs (comparison of independent ROC curves using MedCalc Version 15.6.1 Software bvba, AUC of 0.87 ± 0.02 by MOLLI T1 mapping versus AUC of 0.90 ± 0.01 by ShMOLLI mapping, difference of 0.03 ± 0.02, z statistic 1.34, *P* = 0.17).

Langhans et al. [16] recent looked at the reproducibility of the AAR by T1 and T2 mapping against SPECT in 14 patients with reperfused STEMI at 1.5 T. Although good correlations with SPECT were reported, direct comparison between the two mapping techniques was not performed in terms of per slice correlation, agreement and inter and intra-observer variability.

### Optimal thresholding technique for T1 and T2 mapping

We found that the Otsu thresholding method performed best with excellent inter-observer and intra-observer variability for both mapping techniques. The algorithm automatically divides a signal intensity histogram into two classes requires minimal user input compared to manual delineation, SD thresholding and full width half maximum techniques. It automatically calculates an optimal threshold [22] and has previously been shown to be more accurate and reproducible for quantifying acute MI size by CMR. [25].

### Which to choose: T1 or T2 mapping?

Currently, it would appear that T1 and T2 mapping could be used interchangeably to assess the AAR in reperfused STEMI patients. In studies investigating post-contrast T1 and extracellular volume fraction in acute myocardial infarction patient, there is the possibility of shortening the scanning time by omitting T2 maps as the T1 maps would be available for AAR quantification. However, T1 mapping may not be suitable in acute myocardial infarction patients who also have a chronic infarct in the remote myocardium. T1 mapping CMR has recently been shown to identify chronic infarct with high diagnostic accuracy [26] in a canine model, and using this technique in these patients would require taking into account areas of chronic infarct in the remote myocardium. Our study excluded patients with previous infarct and therefore we cannot comment on the performance of T1 mapping over T2 mapping in patients with co-existing chronic infarcts. From an MR physics point of view, these two techniques are assessing different properties of the myocardium and may explain the limit of agreement of around 10 % on a per-slice comparison and of around 5 % on a per-patient comparison and more work remains to be done to establish the advantage of one mapping sequence over the other.

### Potential future direction

Certain cardioprotective therapies such as ischemic postconditioning [10] and remote ischaemic conditioning (using transient arm or leg ischaemia and reperfusion) [11, 12] has been shown not only to reduce MI size, but also the extent of edema delineated by T2 mapping and T2-weighted imaging leading to an underestimate of the AAR in reperfused STEMI patients. Whether the AAR delineated by T1 mapping is also affected by these therapeutic interventions needs to be investigated. It is currently believed that the AAR delineated by T2-weighted imaging is maximum and constant within the first week following an acute myocardial infarction [27, 28]. Whether the T1 signal remains stable for a longer period of time remains to be tested. A recent pre-clinical study using a porcine model of acute MI has suggested that myocardial edema delineated by T2 CMR may vary over the first week with 2 phases of edema [29]. Whether this is present in reperfused STEMI patients and whether it is apparent with T1 mapping is unknown.

### Limitations

Although, we only included a small number of patients, we did include a range of ischemic times and performed detailed slice-per-slice comparisons, including inter and intra-observer performance. Unfortunately we did not recruit any patients with a circumflex territory myocardial infarction as it would have been interesting to assess the effect of off-resonance artifacts in the lateral wall in those patients with lateral wall MI on both mapping techniques. Our study was performed at 3T and whether T1 mapping performs as well as T2 mapping in quantifying the AAR at 1.5 T in the clinical setting remains to be determined.

## Conclusions

We have shown for the first time that T1 mapping CMR at 3T can accurately quantify the AAR delineated by T2 mapping in reperfused STEMI patients. However, further work is needed to replicate these findings at 1.5 T, assess the dynamic change of T1 over time compared to T2, and determine whether the AAR delineated by T1 mapping is affected by cardioprotective therapies in a similar manner to T2 mapping.

## Abbreviations

PPCI: Primary percutaneous coronary intervention
STEMI: ST-segment elevation myocardial infarction
MI: Myocardial infarction
AAR: Area-at-risk
CMR: Cardiovascular Magnetic Resonance
MOLLI: Modified Look-Locker Inversion Recovery
LGE: Late Gadolinium Enhancement
LV: Left ventricle or ventricular
MVO: Microvascular obstruction
AHA: American Heart Association
ROC: Receiver Operating Characteristic
AUC: Area under the curve
SD: Standard deviation
LAD: Left anterior descending artery
RCA: Right coronary artery
